# Stereotactic Body Radiotherapy (SBRT) of Previously Unirradiated Mucosal Head and Neck Squamous Cell Carcinoma (SCC)

**DOI:** 10.1002/hed.70360

**Published:** 2026-06-29

**Authors:** Mohammed Aldohan, Irene Karam, Amir H. Safavi, Lee Chin, Liying Zhang, Ethan Gooanratne, Madette Galapin, Antoine Eskander, Kevin Higgins, Danny Enepekides, Ian Poon

**Affiliations:** ^1^ Department of Radiation Oncology, Comprehensive Cancer Center King Fahad Medical City Riyadh Saudi Arabia; ^2^ Odette Cancer Centre ‐ Sunnybrook Health Sciences Centre University of Toronto Toronto Ontario Canada

**Keywords:** local failure, medically unfit, stereotactic body radiation therapy, toxicity stereotactic body radiotherapy (SBRT) of unirradiated mucosal head and neck squamous cell carcinoma (SCC), untreated head and neck cancer

## Abstract

**Purpose:**

To evaluate the efficacy and safety of stereotactic body radiation therapy (SBRT) in previously untreated primary mucosal head and neck cancer (HNC) patients unfit for conventional radiation therapy.

**Materials and Methods:**

Retrospective analysis of previously unirradiated primary SCC mucosal HNC patients treated with SBRT from 2011 to 2022.

**Results:**

HN SBRT was prescribed to 74 patients (median age: 81 years [52–97]; 85% ECOG 2–3) with 77 primary tumors (62% oral cavity; 64% T3‐4 disease). A five‐fraction prescription ranging from 35 to 50 Gy (95% received ≥ 40 Gy, 59.7% received ≥ 45 Gy) was delivered twice weekly, with the majority (81%) completing treatment within 14 days and all patients completing treatment. Symptomatic relief was achieved by 84%, including 27.3% with complete symptomatic response. With a median follow‐up of 7.3 months, 1‐year outcomes were 81% local control, 16% regional recurrence rate, and 12% distant metastasis rate. Median progression‐free and overall survival were 6.4 and 8 months, respectively. Acute Grade 3+ toxicity occurred in 36%, primarily mucositis (30%). Late Grade ≥ 3 toxicity was observed in 10% (*n* = 7), including osteoradionecrosis in 5 patients, with residual or concurrent disease present in 5% (*n* = 4). PTV > 159 cm^3^ was significantly correlated with late grade ≥ 3 toxicity (OR = 15.98; *p* = 0.0041).

**Conclusion:**

High‐dose SBRT may deliver high rates of local control and palliation for primary mucosal HNC patients unfit for conventional radiation, but prospective, comparative, high‐quality data are needed. A randomized trial (NCT06641791) comparing SBRT to conventional palliative RT in the elderly unfit HNC patient is underway.

## 1 Introduction

Overall survival (OS) for human papillomavirus‐negative, locally advanced head and neck cancer (LAHNC) remains poor. Treatment outcomes in LAHNC have improved with better surgical techniques and treatment intensification of radiotherapy (RT) with the addition of chemotherapy (CRT) and altered fractionation (AF) [1, 2]. However, the improvement in OS in LAHNC patients was primarily obtained in fit, younger patients, likely due to treatment‐related complications in the elderly (> 70 years old) [3, 4, 5]. Surgical series of well‐selected, elderly patients also report an 8% perioperative mortality and a 43% complication rate [5].

Therefore, there is an unmet therapeutic need for elderly, less fit patients with LAHNC. Among conventional palliative treatment options, palliative (P)RT is often a less intense, short course of RT intended to alleviate consequential symptoms such as pain, bleeding, dysphagia, and facial disfigurement. PRT often provides symptom relief and tumor response but is short‐lived and limited. Systemic palliative options are an option, although they have the potential of severe toxicity in the frail elderly LAHNC, poor and delayed symptom relief, and relatively low response rates [6, 7].

There is limited literature that suggests SBRT may be beneficial in HNC [8, 9, 10]. The SBRT literature for unirradiated primary mucosal HNC, while sparse and heterogeneous in dosing regimens, does suggest that SBRT may deliver effective treatment, symptom relief, and durable local control in a short RT course that can be tolerated by frail, poor performance status (PS) patients [11, 12, 13] with acceptable toxicity. In this report, we present a retrospective analysis from a single institution, examining the treatment results of high‐dose SBRT in 74 patients diagnosed with primary mucosal squamous cell carcinoma (SCC) of the HN who were deemed unfit for radical RT.

## 2 Materials and Methods

### 2.1 Patient Selection

This study, approved by our institution's research ethics board, is a single‐center, retrospective review of patients diagnosed with primary SCC mucosal HNC who underwent SBRT treatment (35–50 Gy in 5 fractions) between 2011 and 2022. All cases were discussed in a multidisciplinary tumor board meeting. SBRT was proposed for patients deemed unsuitable for radical, curative‐intent treatment and unsuitable for systemic therapy. Patient staging followed the 8th edition of the American Joint Committee on Cancer (AJCC) manual [14]. Tumor evaluation was conducted using computer tomography (CT) or magnetic resonance imaging (MRI), with biopsies confirming SCC. Patient characteristics, such as the most common presenting symptoms, stage, Eastern Cooperative Oncology Group (ECOG) PS, dosimetry, and outcome data, were extracted from electronic medical records. Patients with non‐SCC histologies, skin cancer, parotid tumors, or recurrent, previously irradiated tumors were excluded.

### 2.2 Treatment Characteristics

Patients underwent contrast‐enhanced CT simulation, immobilized using a five‐point thermoplastic shell. Concurrent MRI simulation, fused to aid in soft tissue target delineation, was standardly used. All MR simulation images were reported by a diagnostic neuroradiologist to maximize target definition accuracy. Gross tumor volume (GTV) was delineated on the combined MRI–CT scans with T1+ gadolinium, T1 with fat saturation, and T2 sequences fused. A 3 mm planning target volume (PTV) was added to the GTV to account for setup uncertainty and intrafraction movement. A high‐dose clinical target volume (CTV) was not routinely used although a low‐dose CTV (25 Gy in 5 fractions) may include the immediately adjacent nodal volume. Dosages ranged from 35 to 50 Gy/5 fractions (BED10 range: 59.50–100 Gy), intended to deliver a minimum of 99% coverage of the GTV. The PTV was prescribed to a corresponding lower dose, covering at least 95% of the prescribed dose. The volume receiving 115% of the prescription dose was kept to < 1%. Treatments were standardly delivered twice weekly, spaced 2–3 days apart. Constraints for organs at risk followed institutional policy [11]. Treatment decisions and radiation plans were reviewed in head and neck quality assurance rounds weekly. A typical SBRT plan with images is provided in Figure 1.

**FIGURE 1:**
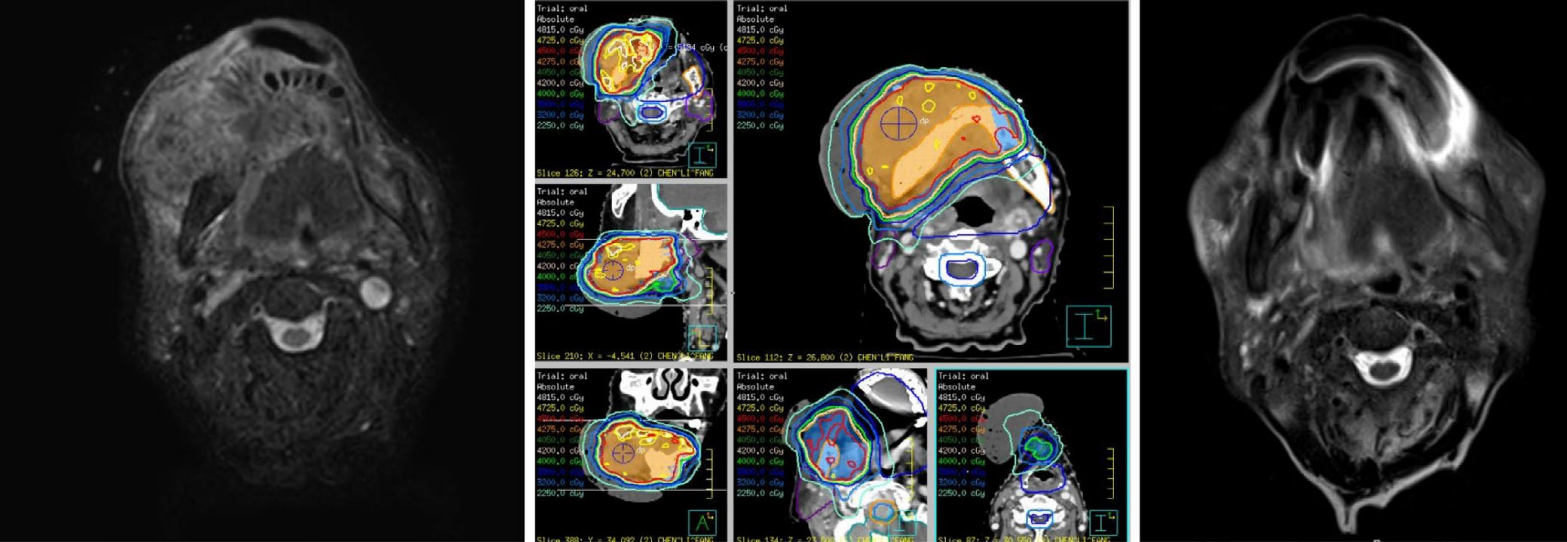
An 88‐year‐old female treated with SBRT (GTV of 45 Gy and PTV of 40 Gy). The pretreatment MRI (left) shows a large, irregular T4N2 SCC mass of the right oral cavity. The posttreatment MRI (right) taken 10 months after receiving 45 Gy in 5 fractions shows significant tumor reduction and only posttreatment related changes. No evidence of disease at 18 months. [Color figure can be viewed at wileyonlinelibrary.com]

RT was delivered via intensity‐modulated radiotherapy (IMRT) or volumetric‐modulated arc therapy (VMAT) on linear accelerators (Elekta Infinity or Versa HD with Agility collimators) with a Hexapod (Elekta AB) robotic couch and was planned on the Pinnacle 3 treatment planning system (Phillips Medical Systems, Madison, WI, USA). Kilovoltage cone‐beam CT (CBCT) was performed daily before treatment. The radiation oncologist was present at a minimum at the first fraction to confirm accurate setup and for CBCT review. Follow‐up of these patients included clinical and imaging assessments performed approximately every 2–3 months following treatment.

### 2.3 Outcomes and Statistics

Patient and clinical characteristics were compiled for all patients and the number of tumors treated. Medians and ranges were used for continuous variables, and proportions for categorical variables. Kaplan–Meier (KM) plots were constructed to visualize local failure (LF), regional recurrence (RR) within or outside the SBRT field, OS, and progression‐free survival (PFS), each with a 95% confidence interval (CI). Toxicity data were collected according to the Common Terminology Criteria for Adverse Events (CTCAE), version 5.0 grading [15].

LF was measured per GTV target and defined as the time from the final SBRT fraction to the clinic visit confirming LF within the GTV volume. The cumulative incidence of LF was determined using the KM method, considering death as a competing risk. RR was measured on a per‐patient level, defined as the interval from the final SBRT fraction to the clinic visit confirming regional/nodal recurrence within the HN. RR was further divided into RR within and outside the PTV volume for separate analysis.

PFS was marked from the last SBRT fraction to any recurrence or death or to the last follow‐up, whichever was earlier. OS was defined as the interval from the final SBRT fraction to the date of death or to the last follow‐up. Tumor response to treatment was retrospectively evaluated on imaging and scored based on the response evaluation criteria in solid tumors (RECIST), version 1.1 guideline [16]. If a patient was symptomatic at presentation, the response was categorized into complete response (CR) for complete resolution of symptoms, partial response (PR) for some symptomatic improvement, or no response (NR) for no symptomatic improvement based on the patient's use of pain medication or reported pain score posttreatment.

A univariate Cox proportional hazard model was employed to identify significant predictive factors associated with LF and PFS. The analyzed potential predictive factors included age, sex, ECOG PS, pre‐SBRT T‐stage (T3‐4 vs. T1‐2), treated subsite, GTV or PTV volume, primary tumor size, mean GTV or PTV dose > 40 versus ≤ 40 Gy, and SBRT dose in PTV or GTV > 40 versus ≤ 40 Gy. Variables with *p* < 0.10 in the univariate analysis were selected for backward stepwise selection in a multivariable Cox proportional hazards regression analysis. The final model incorporated only variables with *p* < 0.05. The analysis was conducted using Statistical Analysis Software (SAS version 9.4; Cary, NC, USA) and the R package (v4.0.3).

## 3 Results

Median patient age was 81 years, ranging from 52 to 97 years. In 74 patients, 77 total lesions were treated with biopsy‐proven SCC histology from October 2011 to June 2022 with a median follow‐up duration of 7.2 months. T4 disease was the most common, present in 43.2% (*n* = 32) of the cases, followed by T3 disease in 20.2% (*n* = 15). Nodal disease was identified in 62.2% of patients (*n* = 46) with the oral cavity as the predominant subsite, accounting for 62% of cases. The majority of patients had an ECOG PS ≥ 2 (*n* = 64 patients, 86%), and 6 (8%) patients were hospitalized during RT. Further patient and treatment characteristics are detailed in Tables 1 and 2.

**TABLE 1:** Patient characteristics.

	Total patients (*N* = 74)
Demographics	
Age	
Median (range)	81.0 (72.0, 88.0) year
Gender	
Female	32 (43.2%)
Male	42 (56.7%)
ECOG categories	
Unknown	2 (2.7%)
1	8 (10.8%)
2	48 (64.8%)
3	15 (20.3%)
4	1 (1.4%)
Predominant symptom at time of presentation pre‐SBRT	
Pain	30 (40.5%)
Mass	29 (39.2%)
Dysphagia/odynophagia	10 (13.5%)
Headache	1 (1.4%)
Bleeding	1 (1.4%)
Asymptomatic	1 (1.4%)
Stridor	1 (1.4%)
Unknown	1 (1.4%)
Tumor site	
Oral cavity	46 (62.2%)
Oropharynx	14 (18.9%)
Hypopharynx	6 (8.1%)
Larynx	4 (5.4%)
Sinonasal	4 (5.4%)
T‐stage	
T0	1 (1.4%)
T1	3 (4.1%)
T2	13 (17.6%)
T3	15 (20.3%)
T4	32 (43.2%)
N_stage	
N0	30 (40.5%)
N1	10 (13.5%)
N2	31 (41.9%)
N3	3 (4.1%)
TNM stage	
Stages I–II	8 (10.8%)
Stage III–IV	66 (89.2%)
SBRT indications	
Declined conventional treatment	4 (5.4%)
Unfit	67 (90.5%)
Hospitalization during RT	
No	68 (91.9%)
Yes	6 (8.11%)

**TABLE 2:** Treatment characteristics.

	Total lesions (*N* = 77)
GTV (cm^3^)	
Mean	62.1
Median (min, max)	61.6 (1.9, 197.8)
PTV (cm^3^)	
Mean	112.9
Median (min, max)	93.7 (3.8, 317.9)
GTV Rx dose (cGy)	
Mean	4212.1
Median (min, max)	4250.0 (3000.0, 5000.0)
SBRT dose	
< 4000	9 (11.7%)
4000	64 (83.1%)
4500	4 (5.2%)
Symptom response	
CR	27 (35.1%)
PR	38 (49.4%)
SD	8 (10.4%)
PD	1 (1.3%)
Unknown	3 (3.9%)
Treatment response	
CR	21 (27.3%)
Mixed	1 (1.3%)
SD	3 (3.9%)
PR	47 (61.0%)
PD	2 (2.6%)
Uknown	3 (3.9%)
Timing of response (months)	
Mean	3.14

Abbreviations: cGy, centigray; CR, complete response; GTV, gross tumor volume; PD, progressive disease; PR, partial response; PTV, planning tumor volume; SBRT, stereotactic body radiotherapy; SD, stable disease.

SBRT doses ranged from 35 to 50 Gy in 5 fractions delivered twice weekly. The median SBRT prescription to the PTV was 40 Gy (median biologically effective dose [BED10] of 72 Gy range: 59.5–100 Gy), and the most common prescription to the GTV was ≥ 45 Gy (*n* = 46, 59.7%). Most patients (*n* = 62, 80.5%) completed their regimen within 14 days, while the remainder (*n* = 12, 19.5%) completed within a range of 9–28 days due to toxicity, all completing treatment. Additional treatment‐related details are provided in Table 2.

At the time of diagnosis, the primary symptoms patients experienced were pain (40.5%), mass effect (39%), and dysphagia or odynophagia (13.5%). Of these patients, 84.4% (*n* = 65) reported symptom relief, with 35% (*n* = 27) confirming complete symptom resolution. Based on the RECIST criteria, 88.3% (*n* = 68) of patients responded to the treatment, with a CR observed in 27.3% (*n* = 21). On average, patients showed a response by the 3‐month mark, with further details presented in Table 2.

On a lesional basis, of the 77 treated lesions, LF occurred in 19.5% (*n* = 15), with a 1‐year cumulative incidence of 19.0% and a 2‐year incidence of 20.7% (Figure 2a). Out of the 74 patients, 14.9% (*n* = 11) experienced an RR. The cumulative incidence was 13% at 6 months and increased to 16.3% by 1 year (Figure 2b). Of the 74 patients, 4 (5.4%) had RR within the GTV, and 7 (9.5%) experienced RR outside of the GTV. Distant metastases (DMs) developed in 8 (10.8%), with a cumulative incidence of 8.8% at 6 months, and 12.1% at both 1 and 2 years (Figure 2c). The median OS was 7.9 months (95% CI, 6.41–10.32), with a 1‐year OS rate of 36% (Figure 2d). The median PFS was 6.4 months (95% CI, 4.70–8.54), with a 1‐year PFS of 30% (Figure 2e).

**FIGURE 2:**
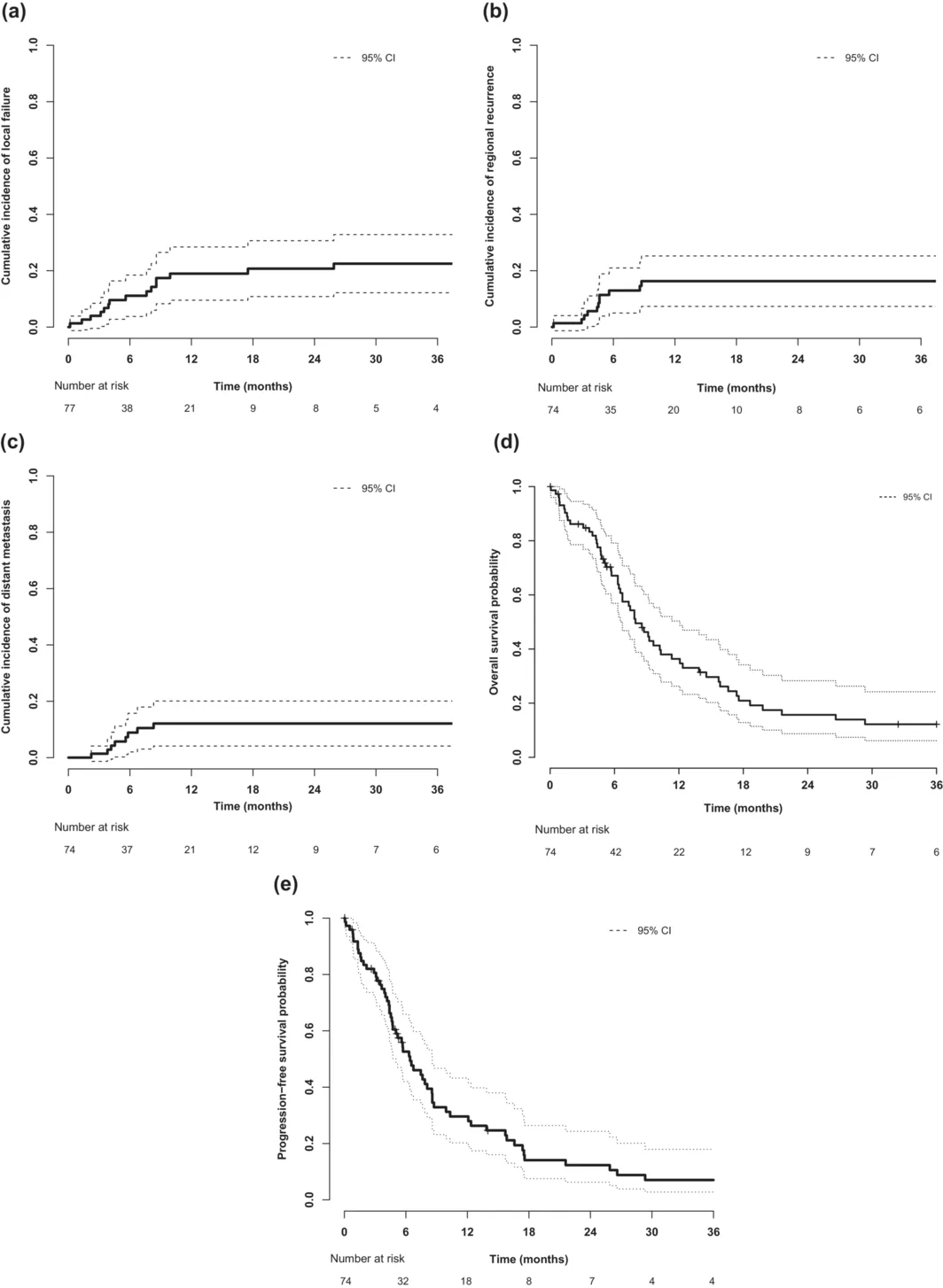
Kaplan–Meier and cumulative incidence curves for oncologic outcomes following SBRT, each shown with a 95% confidence interval. (a) Cumulative incidence of local failure (per lesion; *n* = 77), with death considered as a competing risk. (b) Cumulative incidence of regional recurrence (per patient; *n* = 74). (c) Cumulative incidence of distant metastasis (per patient; *n* = 74). (d) Kaplan–Meier estimate of overall survival (per patient; *n* = 74). (e) Kaplan–Meier estimate of progression‐free survival (per patient; *n* = 74).

In our cohort of 74 patients, 36.5% (*n* = 27) experienced acute toxicity of Grade 3 or higher, while 9.5% (*n* = 7) reported late toxicity of Grade 3 or higher. Specifically, osteoradionecrosis of Grade 3 was diagnosed in five patients (7%); one patient experienced Grade 3 dysphagia, and another patient had severe Grade 5 dysphagia. Four patients (5.4%) who developed Grade 3 or higher toxicity had residual or progressive disease (Table 3).

**TABLE 3:** Toxicity.

	Total patients (*N* = 74)
Acute toxicities	
Acute toxicity	
0	12 (16.22%)
1	6 (8.11%)
2	29 (39.19%)
3	27 (36.49%)
Late toxicities	
Late toxicity grade ≥ 3	
No	67 (90.54%)
Yes	7 (9.46%)
Late toxicity details	
Grade 1–2 toxicities	12 (16.22%)
G3 ORN	2 (2.70%)
G3 ORN (residual disease)	2 (2.70%)
Grade 3 dysphagia (residual disease)	1 (1.35%)
Grade 3 ORN and oropharyngeal ulcer	1 (1.35%)
Grade 5 dysphagia (progressive disease)	1 (1.35%)

A subgroup analysis was performed to identify predictive factors for acute or late Grade 3 toxicity or above, using logistic regression analysis (*n* = 74). The MV analysis revealed that ECOG PS of 3–4 (OR = 4.37; *p* = 0.0184) or higher BED10 values (OR = 1.09; *p* = 0.0367) were more likely to develop acute toxicity of Grade 3 or higher. For late toxicity, MV analysis showed that patients with larger PTVs were more likely to develop late toxicity of Grade 3 or higher (OR = 10.98; *p* = 0.017) with a cut‐off value of 159 cc.

## 4 Discussion

Several PRT strategies have been explored for patients with advanced head and neck cancer who are unsuitable for definitive treatment, with the QUAD shot regimen representing one of the most commonly used approaches [17, 18]. In the original Phase II study by Corry et al., objective tumor response was observed in approximately 53% of patients, with a median OS of 5.7 months. Subsequent series, including that by Lok et al., have demonstrated palliative response rates of approximately 60%–65% and median OS of 5–7 months.

In comparison, the present SBRT cohort demonstrated higher rates of both symptomatic and radiographic response, with 84% of patients reporting symptom improvement, 88% achieving radiographic response, and 27% achieving CR. One‐year local control was 81%, and median OS was 7.9 months. These findings suggest that SBRT may offer a more intensive local treatment strategy delivered over a short course, with the potential for improved tumor response and symptom relief compared to conventional palliative regimens.

However, these comparisons must be interpreted with caution, as QUAD shot studies often include heterogeneous patient populations, including recurrent, metastatic, and previously irradiated disease, whereas the present study focuses on previously unirradiated primary mucosal HNSCC. In addition, QUAD shot regimens are typically delivered over multiple cycles, whereas SBRT is completed in a single short course. While QUAD shot may be associated with lower reported rates of high‐grade toxicity, SBRT may provide a higher biologically effective dose to gross disease, potentially translating into improved local control in selected patients.

Taken together, SBRT may represent a practical alternative in medically unfit patients where rapid symptom relief and durable local control are prioritized.

In Canada, the population aged 85 and older is growing rapidly, with a tripling of Canadians > 85 and older between 2021 and 2050 (2.5 million) [19]. AHNC treatment continues to improve outcomes through treatment intensification schemes that exclude the frail patient. HNC is mostly a disease of the aged, with up to 40% of HNC patients 70 years or older [1] at presentation. While underlying comorbidities and frailty are competing risks that may preclude standard radical treatment of surgery/(C)RT/immunotherapy (IO) to maximize LC with this group of frail, unfit LAHNC patients, cancer progression within the head and neck often remains the most immediate threat to quality and quantity of life [20].

HN SBRT may represent a treatment option to deliver a more durable symptom and local control within a treatment scheme that frail HNC patients can tolerate. This study describes a homogeneous dataset of the largest cohort of unirradiated primary mucosal HNC SCC undergoing SBRT. A comparison with recent studies [21, 22] on SBRT for mucosal HNSCC is listed in Table 4.

**TABLE 4:** Characteristics and results of the most recent reported radiotherapy studies on SBRT for mucosal head and neck squamous cell carcinoma (SCC) compared with the present study.

Author and year	*n*	Study design	Dose in (Gy/Fr)	Median follow‐up months	LC within PTV in 12 months	OS in 12 months	LRR outside SBRT volume in 12 months	Objective SR	Late G3 toxicity
Gogineni 2020 [22]	44	Retrospective	35–40/5	10.5	73%	64%	27%	—	None
Al‐Assaf 2020 [21]	48	Retrospective	40–50/5	15	85.8%	—	11%	84%	G4: 2%
Present study	74	Retrospective	35–50/5	7.2	81%	36%	16.3%	84.4%	G3: 7.8% G5: 1.4%

The local control rate of 81% at 1 year our group achieved with SBRT in an elderly (median age 81 years), frail (ECOG 2/3—85%) cohort, where most cases (64%) were T3/T4 and 61% N+, appears favorable to other palliative regimens, although a direct comparison may be affected by bias. The MVA did not identify any treatment or patient factors that influenced LF, which may be limited by the relatively small sample size and patient mix. Beyond LC, 84% of patients reported objective improvements in symptoms, and 35% reported complete symptom resolution. Notably, 60% of patients received prescription doses ≥ 45 Gy (BED10—85.5), and 95% received ≥ 40 Gy (BED10—72). In a randomized study, SC‐24 showed that symptom control is dose‐dependent in spine bony metastases [23]. Although in a randomized trial comparing moderate dose escalation (20 vs. 30 Gy in 5 daily fractions) [24] in the palliative nonmetastatic AHNC, there were no significant differences in PFS, OS, or quality of life measures.

We observed a 10% rate of late Grade 3 or greater toxicity, which may be attributed to our larger patient sample and the high incidence of advanced disease cases when compared to previously published literature [12, 22]. Upon expert review by three radiation oncologists (M.A., I.K., I.P.) specializing in HNC, the following was noted: The patient with Grade 5 dysphagia exhibited progressive disease and declined a PEG tube placement. Out of the five reporting Grade 3 osteoradionecrosis, three displayed signs (biopsies were not performed) of residual disease, potentially clouding ORN findings. Also, one patient experiencing Grade 3 dysphagia showed signs of recurrence. The remaining three (4%) presented with toxicities in the absence of evidence for disease recurrence. Late toxicity was correlated to PTV size with a statistically significant cut‐off value defined at 159 cc. Future studies to define other late toxicity risk factors will be valuable. The MV analysis correlated increasing age to LF (HR = 1.09; *p* = 0.0.004) which has been previously reported with the frailer HNC patient. We did not identify any variables affecting local control, including dose and T‐stage, although the MV analysis may have been limited by a relatively small patient cohort.

Given the palliative intent of treatment in this population, identifying an optimal therapeutic window that balances symptom control with treatment‐related toxicity is of particular importance. While a higher dose was associated with increased rates of Grade ≥ 3 toxicity, it remains unclear whether dose escalation confers additional palliative benefit. Lower dose regimens such as 35 Gy in 5 fractions may therefore provide meaningful symptom relief with reduced toxicity; however, this hypothesis requires validation in prospective studies.

An important limitation of this study that should be acknowledged is its retrospective nature and the relatively short median follow‐up period of 7.2 months. Also, systemic therapy including IO in this elderly, frail group (ECOG 2/3—85%) was not offered due to toxicity concerns, which may affect these reported outcomes. In addition, the study was conducted in a single institution, and results might not be generalizable to different populations or healthcare settings. With OS as the primary endpoint, the Canadian Cancer Trials Group (CCTG), with funding from the Canadian Institute of Health Research, HN‐13 (NCT06641791), is accruing previously unirradiated, nonmetastatic AHNC patients, randomizing to HN SBRT versus standard PRT across Canada.

## 5 Conclusion

In the palliative setting, high‐dose SBRT may offer an effective regimen for local control and symptom palliation to the primary mucosal AHNC patient who is unsuitable for definitive strategies. Randomized data are required to determine the comparative efficacy of this approach over conventional palliative RT.

## Conflicts of Interest

The authors declare no conflicts of interest.

## Data Availability

The data that support the findings of this study are available from the corresponding author upon reasonable request.
